# A Comprehensive Deep Sequencing Strategy for Full-Length Genomes of Influenza A

**DOI:** 10.1371/journal.pone.0019075

**Published:** 2011-04-29

**Authors:** Dirk Höper, Bernd Hoffmann, Martin Beer

**Affiliations:** Institute of Diagnostic Virology, Friedrich-Loeffler-Institute, Greifswald-Insel Riems, Germany; University of Hong Kong, Hong Kong

## Abstract

Driven by the impact of influenza A viruses on human and animal health, much research is conducted on this pathogen. To support this research, we designed an all influenza A-embracing reverse transcription-PCR (RT-PCR) for the generation of DNA from influenza A virus negative strand RNA genome segments for full-length genome deep sequencing on a Genome Sequencer FLX instrument. For high reliability, the RT-PCRs are designed such that every genome segment is divided into two amplicons and for the most variable segments redundancy is included. Moreover, to minimize the risk of contamination of diagnostic real-time PCRs by sequencing amplicons, RT-PCR does not generate amplicons that are amenable to RT-qPCR detection. With the presented protocol we were able to generate virtually all amplicons (99.3% success rate) from isolates representing all so far known 16 hemagglutinin and 9 neuraminidase subtypes and from an additional 2009 pandemic influenza A H1N1 virus. Three isolates were sequenced to analyze the suitability of the DNA for sequencing. Moreover, we provide a short R script that disambiguates the sequences of the primers used. We show that using unambiguous primer sequences for read trimming prior to assembly with the genome sequencer assembler software results in higher quality of the final genome sequences. Using the disambiguated primer sequences, high quality full-length sequences for the three isolates used for sequencing trials could be established from the raw data in *de novo* assemblies.

## Introduction

Influenza A virus is one of the major threats in modern health care. Besides influenza viruses that cause seasonal epidemics, pandemic viruses such as the 2009 H1N1 swine origin influenza A virus have a significant impact on human and animal health. Therefore, intensive research is carried out tackling different aspects of influenza A virus properties, very often trying to correlate phenotypes with genotypes.

The segmented negative strand RNA genome of influenza A virus comprises 8 segments ranging in size approximately from 850 nt to 2350 nt. Novel viruses arise due to antigenic drift and antigenic shift. Antigenic drift is caused by continuous mutation of the genomes due to the high error rate of the viral RNA-dependent RNA-polymerase. Reassortments of genome segments during infection of a single host with different influenza A viruses result in antigenic shift (reviewed in [1]). Consequently, a high variability of the genome composition leading to diverse virus mixtures may be expected. Therefore, the complete characterization of influenza A viruses is only possible by precise determination of full-length genome sequences by deep sequencing. Also for diagnostics, there is the need for comprehensive tools in this field. This demand has been highlighted once more by the sudden and unexpected emergence and fast spread of the pandemic H1N1 swine origin influenza A virus in 2009.

The existing DNA sequencing capacity that is now available with the different next generation sequencing technologies [2] more than suffices to accurately sequence any DNA sample at any desired sequence depth. On the contrary, adequate protocols for correspondingly satisfying preparation of DNA in sufficient amount and quality for sequencing of complete virus genomes are not at hand.

The most apparent problem in full-length virus genome sequencing is to target the sequencing process away from host nucleic acids to viral nucleic acids. Preparing a simple shotgun sequencing DNA library, the most comprehensive approach, will most likely result in a huge amount of host specific instead of full-length viral sequences. The same holds true when working with libraries prepared from cDNA synthesized from RNA with random priming [3], [4]. So far, different techniques to focus the sequencing have been described [5], [6], [7], [8]. First of all, the most obvious is to enrich virions from cell culture or from host tissue or fluids by centrifugation before extracting the genomic DNA/RNA [5]. This may be a very time-consuming and laborious procedure with sometimes uncertain outcome. Secondly, it is possible to enrich for the portion of nucleic acids that is the aim of the sequencing effort if these can be caught by probing the complete DNA/RNA. This procedure was engaged, for example, in the first official RNA sequencing protocol for the Genome Sequencer FLX (GS FLX) [7]. Here, the influenza A genome segments were enriched by hybridization to biotinylated probes directed against the conserved ends of the genome segments. Subsequently, the probes were bound to streptavidin beads, the samples washed and finally the captured RNA was eluted. A similar approach was used by Monger and colleagues [8] who depleted host nucleic acids by probing total RNA with labeled host nucleic acid. Another approach to target the sequencing to viral nucleic acid was published by Potgieter and colleagues [6]. In this case, a protocol was designed to enrich for double stranded RNA (dsRNA) exclusively representing the bluetongue virus or other dsRNA-virus genomes. Nevertheless, as with the first option, targeting the sequencing effort to a subpopulation of the RNA extracted from a sample also is laborious and the risk of failure is high, as the outcome might not be sufficient in quality or amount to allow for sequencing.

Consequently, it is straightforward to target the sequencing by simply targeting the preparation of DNA for sequencing by specific PCR primers. In the case of highly pathogenic avian influenza A H5N1, it was possible to target and simultaneously speed up the sequencing by a sensitive and reliable reverse transcription-PCR (RT-PCR) [9]. Enrichment of virus nucleic acids by the different available techniques mentioned above was not necessary. Here, we describe a method that fulfills the demand for easy and reliable, but in addition at the same time broad and comprehensive sequencing of influenza A viruses that goes far beyond the previously reported specialized H5N1 sequencing approach.

## Materials and Methods

### Viruses

The virus strains used in this study (see table 1) were obtained from the virus collection of the National Reference Laboratory for Avian Influenza at the Friedrich-Loeffler-Institut, Greifswald, Insel Riems, Germany.

**Table 1:** Virus isolates used in this study.

Type	Virus
H1N1	A/duck/Germersheim/R30/06
H2N2	A/guinea fowl/Germany/DZ3/85
H3N1	A/duck/Germany/R2555/06
H4N6	A/mallard/Föhr/WV1806-09K/03
H5N2	A/mallard/Föhr/1313/03
H6N2	A/turkey/Germany/617/07
H7N3	A/duck/Italy/636/03
H8N4	A/turkey/Ontario/6118/68
H9N2	A/duck/Germany/113/95
H10N7	A/chicken/Germany/N/49
H11N6	A//Germany/R2795/06
H12N5	A/duck/Alberta/60/76
H13N8	A/Black-headed gull/Mukran/R2613/06
H14N5	A/mallard/Gurirv/263/82
H15N9	A/shearwater/West Australia/2576/79
H16N3	A/herring gull/Mukran/R2788/06
H1N1 pandemic	A/Germany-BY/74/2009

### RNA extraction

Total RNA was isolated from allantoic fluid with Trizol LS (Invitrogen, Darmstadt, Germany) in combination with RNeasy Mini spin columns (Qiagen, Hilden, Germany). First, 250 µl of allantoic fluid were mixed with 750 µl Trizol LS and processed as recommended by the manufacturer until phase separation. Instead of RNA precipitation, the RNA containing aqueous phase was then mixed with ethanol (final concentration ≥40% (vol/vol)) and loaded onto an RNeasy Mini spin column. Subsequently, the RNeasy Mini protocol was followed until elution of RNA. Alternatively, total RNA was isolated from allantoic fluid (140 µl) with a QIAamp viral RNA minikit (Qiagen, Hilden, Germany), according to the manufacturer's instructions.

### PCR, library preparation, sequencing, and sequence assembly

DNA was generated either in a one-step or in a two-step RT-PCR. For the one-step RT-PCR protocol, cDNA was generated from total RNA diluted 10-fold in RNA-safe buffer (50 ng/µl carrier RNA, 0.05% Tween 20, 0.05% sodium azide in RNase-free water) [10] by RT-PCR with a SuperScript III one-step RT-PCR system with Platinum Taq high-fidelity polymerase (Invitrogen). For every complete influenza A genome, 16 PCRs were set up with the primer combinations listed in table 2. Five microliters of the dilute template and 1 µl of each primer (10 µM; final concentration in the PCR mixture, 0.4 µM) were mixed and denatured for 2 min at 95°C. Immediately after denaturation, the samples were frozen in liquid nitrogen, and subsequently, 18 µl of the PCR master mixture (12.5 µl 2× reaction mixture, 1 µl Superscript III reverse transcriptase-Platinum Taq high-fidelity polymerase enzyme mixture, 4.5 µl RNase and DNase-free distilled water) was added. The RT-PCR was performed in a model 2720 thermal cycler (Applied Biosystems, Darmstadt, Germany). The cycling parameters were 30 min at 50°C; 2 min at 94°C; 40 cycles of 15 s at 94°C, 30 s at 50°C, and 3 min at 68°C; and 1 cycle of a hold for 5 min at 68°C. Alternatively, two-step RT-PCR was performed with SuperScript VILO cDNA Synthesis Kit (Invitrogen) and AccuPrime Taq DNA Polymerase High Fidelity (Invitrogen). For the two-step protocol, 2 separate reverse transcriptions were performed, one for the 5′ parts and one for the 3′ parts of the segments. For this purpose, 5 µl total RNA were mixed with 4 µl 5× VILO reaction mix, 2 µl 10× Superscript enzyme mix, and 7 µl RNase and DNase-free distilled water. To this mix containing random hexamer primers, for the 5′ portion of the segments 2 µl of general primer pan-IVA-1F-M13F (10 µM) or for the 3′ portion of the segments 2 µl of RNase and DNase-free distilled water were added. Reverse transcription was then performed in a model 2720 thermal cycler (temperature profile: 10 min at 25°C, 5 min at 42°C, 30 min at 55°C, 5 min at 60°C, 5 min at 85°C, and finally 15 s at 10°C). Immediately after reverse transcription, PCR was set up. To this end, 2.5 µl of the cDNA were used as template and combined with 2.5 µl 10× AccuPrime PCR Buffer 1, 0.1 µl AccuPrime Taq High Fidelity, 17.9 µl RNase and DNase-free distilled water, and 2 µl of a primer mix containing primers (each 5 µM) for amplification of the respective portion of the genome segment according to table 2. The reaction mixes were incubated initially 1 min at 94°C; 40 cycles of 15 s at 94°C, 30 s at 50°C, and 3 min at 68°C; and 1 cycle of a hold for 5 min at 68°C. The PCR products both from the one-step and the two-step protocol were purified with Ampure Beads (Beckman Coulter Genomics, Bernried, Germany) according to the manufacturer's instructions. The purified DNA was quantified with a model 1000 spectrophotometer (NanoDrop Technologies, Inc., Wilmington, DE) in the double-stranded DNA mode and was then pooled in equimolar amounts. This DNA pool (800 ng to 3 µg) was transformed to DNA libraries by using a GS DNA library preparation kit (Roche, Mannheim, Germany) using the protocol of Wiley and colleagues [11] with the modifications of Leifer and colleagues [12]. For bead bound clonal amplification of the DNA libraries, the DNA libraries were subjected to duplicate emulsion PCRs (emPCR) with the GS emPCR kit I (Roche), according to the manufacturer's instructions, with 2 copies per bead. After bead recovery and enrichment, the beads were sequenced using a GS LR70 sequencing kit (Roche) and the appropriate instrument run protocol. The resulting sequencing reads were sorted according to the genome segments to which they related and were subsequently assembled into one contig (i.e., a set of overlapping sequencing reads) per segment with the GS FLX sequence assembly software newbler (version 2.3; Roche). During the assembly, the primer sequences were trimmed off the raw data. In addition, to check whether or not the sequences were complete, we performed a mapping along appropriate reference sequences using the GS FLX reference mapper software (version 2.3; Roche).

**Table 2:** Primers used for RT-PCR amplification of influenza A virus genomic segments.

	Genome segment	Primer designation	Primer sequence	Approximate product size (bp)
5′ part	general forward	Pan-IVA-1F_M13F	TCCCAGTCACGACGTCGTAGCGAAAGCAGG	
	1	Pan-IVA-S1-1254RPan-IVA-S1-1395R	GCA ATC CTC YTG TGA RAA YAC CAT ATT GTC RAT RGG TTC AAT TCC CCA	12701415
	2	Pan-IVA-S2-1253R	GAT TGG AGY CCR TCC CAC CA	1370
	3	Pan-IVA-S3-1264RPan-IVA-S3-1311R	CCT TGT TGA ATT CAC TYT GDA TCC A YCC TAT YTC ATC AAG YTC TAT CCA	12801330
	4	Pan-IVA-S4-1451RPan-IVA-S4-1565R	ACC AGA AGT TCA GCA TTR TAW GWC C AAA CAA CCA TTN CCN ATN TCC TTN GCA TT	13851495
	5	Pan-IVA-S5-1471R	CGA TCG GGY TCG BTG CCT T	1490
	6	Pan-IVA-S6-1158RPan-IVA-S6-1351R	TNT TGC TTW TNG TTC TTC CYA WCC A CCT CTD ATC AAC TCH ACM YAR AAR CA	10751265
	7	Pan-IVA-S7-124R	TGC AAA AAC ATC TTC AAG TYT CTG	140
	8	Pan-IVA-S8-600RPan-IVA-S8-635R	CTC GAA CHG TGT TAY CAT TCC A GNT TCT CCA AGC GAA TCT CTG TA	610645
3′ part	general reverse	Pan-IVA-1R_M13R	GGAAACAGCTATGACCATGAGTAGAAACAAGG	
	1	Pan-IVA-S1-1051FPan-IVA-S1-1102F	GAA GAA GTG CTT ACR GGC AAY CT GGR TAT GAR GAR TTC ACA ATG GT	13101260
	2	Pan-IVA-S2-1233F	TGA GYC CTG GRA TGA TGA TGG	1130
	3	Pan-IVA-S3-957FPan-IVA-S3-912F	GAG AAC ATT CTT TGG ATG GAA RGA TCA TGA GGG RGA GGG RAT ACC	12951340
	4	Pan-IVA-S4-1157FPan-IVA-S4-1198F	CTA TTT GGA GCH ATH GCN GGT TTY AT GGA ATG RTA GAT GGT TGG TAY GG	730685
	5	Pan-IVA-S5-1448F	GGG AGT CTT CGA GCT CTC	155
	6	Pan-IVA-S6-866FPan-IVA-S6-906F	CAY TWW GAG GAR TGC TCC TGY TA TCA NAT GTG TNT GCA GRG AYA A	635595
	7	Pan-IVA-S7-76F	AGG CCC CCT CAA AGC CGA	970
	8	Pan-IVA-S8-526F	ATA CTV ATG AGG ATG TCA AAA ATG C	375

## Results

### PCR Design

**Figure 1:**
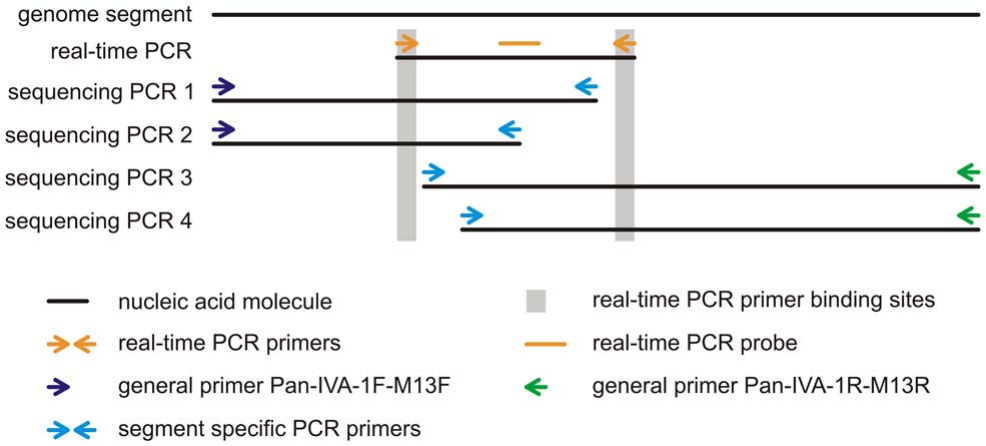
Schematic of the primer localizations. PCR products used for sequencing are designed such that every segment is amplified in two amplicons. Moreover, no amplicon containing the complete region necessary for detection in a diagnostic real-time RT-PCR is generated. This reduces the risk of compromising nucleic acid diagnostics by sequencing amplicons. For some segments, redundancy is introduced for more reliability.

Primers were designed on the basis of alignments of all influenza A full-length segment sequences that included the conserved ends and regardless of the host of the virus isolate available at the time of primer design. Positioning of the primers for reverse transcription and amplification was done following three key criteria: (i) None of the 8 genome segments should be reversely transcribed and amplified as a single amplicon but for better performance be divided in a 5′- and a 3′-part. (ii) In order to reduce the risk of PCR failure caused by non-matching primers due to influenza A virus evolution, we made use of the conserved regions at both the 5′ and the 3′ segment ends as primer binding sites. (iii) To be also compatible with diagnostic settings, the two amplicons to be generated for each segment should, if possible, not lead to the generation of DNA traceable in the real-time PCR assays in use in diagnostic laboratories for influenza A diagnostics. This prevents interference with diagnostics by PCR products that might be released at any step during the sequencing library preparation process. The RT-PCR design resulting from these considerations is depicted schematically in figure 1. As can be seen in figure 1 (and table 2), in some instances, we use redundant inner segment specific primers. This redundancy is necessary due to the high variability in the sequences of the respective genome segments.

### Generation of sequencing amplicons

The functionality of our PCR protocol was tested with 17 different influenza A isolates that comprise all so far known 16 hemagglutinin (HA) and 9 neuraminidase (NA) types. In addition, a 2009 pandemic H1N1 influenza A virus was included in the panel. Table 3 summarizes the results of the PCRs for all segments from all isolates that were probed. Altogether, 270 out of 272 PCRs (99.3%) that were performed were successful, i.e. with only very few exceptions all amplicons from all isolates could be generated. In no case the PCRs for both the 5′- and the 3′- fragments for one segment failed. Therefore, even in the case of a PCR failure we were able to gain sequence information for the respective genome segment.

**Table 3:** Overview of the PCR results obtained with all different virus isolates.

	5′ part								3′ part							
Subtype\Segment	1	2	3	4	5	6	7	8	1	2	3	4	5	6	7	8
H1N1	+	+	+	+	+	+	+	+	+	+	+	+	+	+	+	+
H1N1 pandemic	+	+	+	+	+	+	+	+	+	+	+	+	+	+	+	+
H2N2	+	+	+	+	+	+	+	+	+	+	+	+	+	+	+	+
H3N1	+	+	+	+	+	+	+	+	+	+	+	+	+	+	+	+
H4N6	+	+	+	+	+	+	+	+	+	+	+	+	+	+	+	+
H5N2	+	+	+	+	+	+	+	+	+	+	+	+	+	+	+	+
H6N2	+	+	+	+	+	+	+	+	+	+	+	+	+	+	+	+
H7N3	+	+	+	+	+	+	+	+	+	+	+	+	+	+	+	+
H8N4	+	+	+	+	+	+	+	+	+	+	+	+	+	+	+	+
H9N2	+	+	+	−	+	+	+	+	+	+	+	+	+	+	+	+
H10N7	+	+	+	−	+	+	+	+	+	+	+	+	+	+	+	+
H11N6	+	+	+	+	+	+	+	+	+	+	+	+	+	+	+	+
H12N5	+	+	+	+	+	+	+	+	+	+	+	+	+	+	+	+
H13N8	+	+	+	+	+	+	+	+	+	+	+	+	+	+	+	+
H14N5	+	+	+	+	+	+	+	+	+	+	+	+	+	+	+	+
H15N9	+	+	+	+	+	+	+	+	+	+	+	+	+	+	+	+
H16N3	+	+	+	+	+	+	+	+	+	+	+	+	+	+	+	+

### Sequencing the PCR products

Most importantly, we were interested in the applicability of the amplicons for sequencing with regard to purity, i.e. absence of non-specifically amplified host sequences, and with respect to completeness of the influenza A sequences. Therefore, we sequenced the DNA fragment pools confected from the PCR products of three isolates. For this sequencing trials, we chose A/turkey/Ontario/6118/68 (H8N4), A/shearwater/West Australia/2576/79 (H15N9), and the 2009 pandemic H1N1 A/Germany-BY/74/2009 (H1N1).

### Primer disambiguation for sequence analysis

In general, in assemblies of data obtained from sequencing PCR products it is necessary to trim off the primer sequences before assembly in order not to distort the final sequences. This is even more important when amplicons have been generated with primers including ambiguities, i.e. incompletely specified bases [13], as is the case with the primers used here. Failure to trim off the primer sequences will result in final sequences which include these ambiguities and hence deviate from the true sequence.

The powerful 454 assembler newbler used in this study cannot handle nucleotide sequence ambiguity codes in trim information [14]. If there is no clearly dominating base at a certain position, the assembler is not able to determine the consensus base at that position. This either leads to low quality basecalls, single base insertions or deletions, or the software splits the sequences at these ambiguous positions leading to a fragmentation of the final genome sequences. Therefore, it is necessary to disambiguate the primer sequences to allow for correct trimming. To this end, a short R [15] program (see supplemental File S1) was written. This program makes use of the seqinr package [16]. Table 4 summarizes the data obtained in *de novo* assemblies with or without primer disambiguation. From the numbers shown there it is evident that disambiguation of the primers enhances final data quality.

**Table 4:** Summary of the impact of primer disambiguation on sequence quality obtained by *de novo* assembly.

	Ambiguous primers			Unambiguous primers		
Isolate	Number of Contigs	Number of bases	Number (%) of Q39− bases^a^	Number of Contigs	Number of bases	Number (%) of Q39− bases^a^
H1N1	23	13814	81 (0.59)	18	13528	49 (0.36)
H8N4	36	13806	84 (0.61)	26	13507	64 (0.47)
H15N9	67	14626	163 (1.11)	51	14301	130 (0.91)

a Q39−-bases are those with a quality score of 39 or less, i.e. with probability ≤99.987%.

Due to the impact of primer disambiguation, for all further analyses, disambiguated primer sequences were used.

### Analysis of data quality, quantity, and usability

For analyses of the quality and quantity of the obtained raw sequences, we first performed a mapping of the reads along the known reference sequences. This allowed us to assess the purity, completeness, and fidelity. Moreover, the raw data were assembled without any advance sequence information in order to test the usability for *de novo* assembly.

#### Reference mapping

The purity of the generated DNA was different for the three isolates. In the worst case, A/shearwater/West Australia/2576/79 (H15N9), the proportion of non-specifically amplified and subsequently sequenced host nucleic acids was 3.7%. In the remaining 2 cases, 3.3% (A/turkey/Ontario/6118/68 (H8N4)) and 1.0% (A/Germany-BY/74/2009 (H1N1)) of all sequencing reads were host sequences.

With regard to completeness, we were particularly curious whether the complete sequences for segments 5 and 7 could be generated. This was interesting because we used very short PCR products (<300 bp) for the 3′ and 5′ part of these segments, respectively. Such short DNA fragments are depleted during preparation of the sequencing libraries. Nevertheless, mapping of the sequence reads against the appropriate reference sequences showed that all segments had been completely sequenced. In all cases, only the sequences of the binding sites of the general forward and reverse primers, respectively, were missing from the final sequences due to primer trimming.

Besides the assessment of DNA purity and sequence completeness, mapping also provided information about the fidelity of the newly generated sequences. The results of the sequence comparisons are compiled in table 5. Except for a few deviations in the regions of the general primers, i.e. outside the coding regions, we rarely detected sequence deviations in the sequences of all 3 isolates. With regard to the deviations in the primer regions it is necessary to note that these occur due to primer trimming. Primer trimming leads to a diminished sequence depth in the trimmed regions causing a diminished sequence quality. With regard to those deviations which were found in the coding regions, we detected 3 differences between newly established and the reference sequences for all segments of isolate A/shearwater/West Australia/2576/79 (H15N9). Namely, we found one deviation in the HA gene (a677g), one base substitution in the NA (a866t), and another difference in the NP gene (a1459c) which is a primer derived substitution. In the sequences for isolate A/turkey/Ontario/6118/68 (H8N4), we detected 1 difference within the open reading frames. This n499g was located in segment 1 and obviously is not a deviation from the reference sequence. The figures for the pandemic H1N1 virus were much the same as for the other 2 isolates. Here, we also found a single deviation from the reference sequences, namely an a837g substitution in segment 2. In summary, we conclude that the sequence fidelity achievable with the established protocol is excellent. With an overall error rate of only 0.032% (including those deviations that are located in the general primer regions) the concordance of the newly determined with the beforehand known reference sequences from all 3 isolates that were sequenced is very high. It is important to note that the deviations may be acquired by the virus during repeated cell or egg culture passages.

**Table 5:** Details of the sequence comparison performed by mapping the generated sequencing reads along the previously reported sequences as a reference.

	H1N1		H8N4		H15N9	
Segment	Reference^a^	Deviations^b^	Reference^a^	Deviations^b^	Reference^a^	Deviations^b^
1	HM138498.1	none	CY005831.1	n499g (X158E)	GU052266.1	none
2	HM138499.1	a837g (silent)	CY014662.1	none	GU052265.1	none
3	HM138497.1	none	CY005830.1	g8-d	CY005410.1	a2220-d, t2230-d,
4	HM138501.1	a18-d	CY014659.1	a4g^c^ ^d^, -1736t^d^	CY006034.1	a677g (Q219R)
5	HM138494.1	a4-d	CY005829.1	none	CY005727.1	a1459c^c^ (T472P)
6	HM138500.1	none	CY014660.1	a4g^c^ ^d^	CY005407.1	a866t (Y283F)
7	HM138496.1	none	CY005828.1	none	GU052261.1	none
8	HM138495.1	none	CY014661.1	none	GU052263.1	c2t^c^ ^d^

a Genbank Accessions of the reference sequences used for the mapping.

b Nucleotide substitutions, insertions, and deletions compared to the reference sequences indicated in the column reference; the impact on the coded amino acid is given in parentheses.

c primer derived deviation.

d mutation outside the coding region.

#### De novo assembly

In order to test if the quality of the raw data and the overlaps of the PCR amplicons are sufficient for a *de novo* assembly, we assembled the raw sequence data without advance sequence information. To this end, we performed an assembly of the raw sequencing reads using the 454 assembler software newbler. As published before for sequencing of highly pathogenic avian influenza A H5N1 viruses [9], complete assembly into a single contig (a set of overlapping sequencing reads) per segment was only possible after allocating the sequencing reads to the segments. The assemblies of the data resulted in one contig per segment for all 3 isolates. Only the sequence for the segment 7 of the H8N4 was truncated. Here, the sequence for nucleotides 1–76 was missing in the *de novo* assembled sequence, caused by the depletion of the small fragment representing this region. Hence, the coding sequence for the H8N4 genome was 99.59% and those for the two other isolates were 100% complete. The main characteristics of all *de novo* assemblies are summarized in table 6. As for mapping, the fidelity of the *de novo* assembled sequences was very high. Here, the total error rate was 0.030%, again including those mutations that were detected in the general primer binding regions which resulted in a relatively low quality due to the trimming during the assembly.

**Table 6:** Summary of data characterizing the quantity and quality of the sequences obtained by *de novo* assembly.

Isolate	Length(no. of bases)	% Coverage of the coding sequence	No. (%)of Q40+ bases
A/Germany-BY/74/2009(H1N1)	13520	100	13458 (99.69)
A/turkey/Ontario/6118/68(H8N4)	13421	99.59	13798 (99.65)
A/shearwater/West Australia/2576/79(H15N9)	13464	100	13223 (99.53)

a Q40+-bases are those with a quality score of 40 or higher, i.e. with probability ≥99.99%.

The results of the comparisons of our *de novo* assembled unrevised sequences with the available reference sequences (table 7) predominantly showed the same differences which were detected by mapping (see table 5). Remarkably, four of the deviations detected by mapping located in the general primer binding regions were not detected in the assemblies. This provides an additional hint that these deviations are artifacts. Moreover, with regard to the coding regions, we did not detect the a866t substitution in the H15N9 NA sequence. On the other hand, three single nucleotide deletions were found in the *de novo* assembled sequences. In particular, we found one deletion in segment 7 of the pandemic H1N1, one deletion in segment 6 of the H8N4 virus, and one deletion in segment 5 of the assembly for the H15N9 virus's sequence. From the detailed assembly characteristics stored by the assembler software, all these deletions could be identified as undercalls, i.e. as artifacts in the assembly. Taken together, despite the differences that are described here, the overall consistency of the unrevised sequences with the reference sequences is 99.97%.

**Table 7:** Details of the comparison of the unrevised *de novo* assembled with the available reference sequences.

	H1N1		H8N4		H15N9	
Segment	Reference^a^	Deviations^b^	Reference^a^	Deviations^b^	Reference^a^	Deviations^b^
1	HM138498.1	none	CY005831.1	n499g (X158E)	GU052266.1	none
2	HM138499.1	a837g (silent)	CY014662.1	none	GU052265.1	none
3	HM138497.1	none	CY005830.1	none	CY005410.1	none
4	HM138501.1	a18-d	CY014659.1	a4g^c^ ^d^, -1736t^d^	CY006034.1	a677g (Q219R)
5	HM138494.1	none	CY005829.1	none	CY005727.1	a1459-
6	HM138500.1	none	CY014660.1	a4g^c^ ^d^, a1327-	CY005407.1	none
7	HM138496.1	c58-	CY005828.1	none	GU052261.1	none
8	HM138495.1	none	CY014661.1	none	GU052263.1	c2t^c^ ^d^

a Genbank Accessions of the reference sequences used for the mapping.

b Nucleotide substitutions, insertions, and deletions compared to the reference sequences indicated in the column reference; the impact on the coded amino acid is given in parentheses.

c primer derived deviation.

d mutation outside the coding region.

## Discussion

In several reports the possibility to benefit from shotgun high throughput sequencing for applications in virus research has been shown (see for instance [5], [6], [7], [8]). However, none of the presented protocols is useful for targeted full-length genome deep sequencing. This is due to limitations in the sample preparation caused by failure to focus or laborious protocols used to target the sequencing process to the aim of the work. Our previously reported protocol for sequencing highly pathogenic influenza A H5N1 [9] is very sensitive, accurate, and useful for the specialized goal. However, its spectrum is too narrow to be useful in influenza A virus research in general or in situations like the 2009 pandemic of the swine origin influenza A H1N1. Therefore, we set out to improve the method making use of the benefits of our first protocol but adding usability and broadening the spectrum of the method to influenza A viruses regardless of the subtype.

The presented results of the sequencing trials done with 3 isolates clearly show that we reached our goal to broaden the spectrum without losing the specificity that is necessary to target the sequencing. With a maximum of only 3.7% of the reads representing host nucleic acids, we obviously successfully direct sequencing to the influenza A genome. Nevertheless, the scale of our approach is very good. This is highlighted by a success rate of about 99.3% for all PCRs as tested with 17 different isolates that represent all 16 HA and 9 NA types, respectively, and one 2009 pandemic H1N1 virus. Therefore, we conclude that focusing the sequencing must not necessarily diminish comprehensiveness. Unlike the protocol for subtype independent sequencing of influenza A viruses initially provided by 454 life sciences for use with the GS FLX [7], we neither need delicate techniques for enrichment of the target nucleic acid nor for preparation of cDNA carrying the adapters necessary for GS FLX sequencing.

By introduction of the protocol of Wiley and colleagues [11] and omission of the gel purification initially included in the sequencing protocol for highly pathogenic influenza A H5N1 [9], we gain the possibility to fully automate sample preparation. Only quantification of the PCR products and preparation of the DNA pools has to be done manually. This permits to raise the throughput which is possible since the introduction of the MID adapters for the GS FLX. These MID adapters provide a recognizable sequence tag at the beginning of each read. These tags allow to group the sequence reads belonging to different DNA libraries. Taken together, this makes sequencing less laborious and more economic.

Another benefit of the new protocol presented here is the design of the PCRs in order not to compromise diagnostic qPCR assays in use in influenza diagnostic laboratories. With the exception of the two small fragments generated for sequencing the M and NP segments, none of the sequencing amplicons is detectable in the respective qPCR assays (data not shown). Therefore, laboratories can be protected from sequencing amplicons that may interfere with real-time PCR diagnostics. Skipping the two small fragments for segments 5 and 7 does not result in loss of much information: In the case of segment 5, the 5′ portion, which is not detectable with qPCR assays, comprises 95% of the coding region. For segment 7, the small 5′ part adds 17 codons, i.e. about 7% of the ORF.

Sequencing RNA directly from specimens without enriching for virus nucleic acids results in high proportions of host sequence reads. In two different studies in which new viruses were identified only 0.014% and 0.091% of the reads, respectively, represented virus sequences [3], [4]. This underlines the necessity to direct the sequencing process. The aforementioned different approaches for focusing the library preparation and hence the sequencing process to the virus nucleic acids are less restricted than our approach [5], [6], [7], [8] but, nevertheless, strong constraints exist: The protocol of Potgieter and co-workers [6] only works with dsRNA, excluding its use for influenza sequencing. The authors do not provide information about the amount of contaminating host reads. With the random cDNA library preparation protocol currently provided by Roche/454 Life Sciences paired with the enrichment protocol of Simons and Hutchison [7], we reached a host sequence contamination level of 87.5%. Moreover, this protocol relies on pre-existing knowledge as does our method. Monger and colleagues [8] report that after de-enrichment of host nucleic acids by hybridization still 84% of the sequencing reads represented host sequences. Due to all these inconveniences, we chose RT-PCR for the targeting. On the one hand, RT-PCR in general is a very robust and sensitive technique which enables us to sequence whole genomes from samples with a broad range of virus content. On the other hand, the focused sequencing results in high amounts of target sequence data. In turn, this renders sequencing with any depth that may be desired economically possible. The huge amount of data enables quasispecies analyses by sequencing every genome position hundreds or even thousands of times. The possibility to benefit from deep sequencing data for quasispecies analysis has already been shown before with African horse sickness virus and classical swine fever virus [6], [12]. With our here presented protocol, this kind of analyses is made possible for all subtypes of influenza A viruses.

Altogether, we were able to markedly improve our previously reported protocol [9]. With the high quality of the final sequence data that are produced with our technique, we conclude that the presented method is suitable for routinely sequencing complete influenza A virus genomes with a high throughput. In addition, due to the great sequence depth that can be achieved even quasispecies analysis at the full genome level is made possible.

## Supporting Information

File S1 This file contains the R-code that disambiguates nucleotide sequences and writes a new file containing these disambiguated sequences. The script relies on the seqinr package [16].
(PDF)
