# Supplementary material for: A Comprehensive Deep Sequencing Strategy for Full-Length Genomes of Influenza A
Source: PLoS One. 2011 Apr 29;6(4):e19075. doi: 10.1371/journal.pone.0019075 (PMC3084732; doi:10.1371/journal.pone.0019075)
Supplement: File S1 — This file contains the R-code that disambiguates nucleotide sequences and writes a new file containing these disambiguated sequences. The script relies on the seqinr package [16]. (PDF) [file pone.0019075.s001.pdf]

```
################### R-script to disambiguate DNA sequences ###################
# This script reads in a given FASTA formatted sequence file and translates all
# incompletely specified bases (see Nomenclature Committee of the International
# Union of Biochemistry. (1986) Nomenclature for incompletely specified bases in
# nucleic acid sequences. Recommendations 1984. Proc Natl Acad Sci U S A 83:4-8)
# into the single bases coded by these ambiguity codes. As a result, a new FASTA
# formatted file is written containing multiple copies of the provided original
# sequences representing all possible combinations of unambiguous sequences.
################################################################################


require(seqinr)     # load required package

rm(list = ls())     # clean up the workspace

code = c("y", "r", "w", "s", "k", "m", "b", "d", "h", "v", "n", "x")    # generate a vector of possible ambiguity codes
anzVar = c(2, 2, 2, 2, 2, 2, 3, 3, 3, 3, 4, 4)    # generate a vector holding the number of possible translations for
each of the ambiguity codes in code
ambinuc <- data.frame(cbind(code, anzVar, a = FALSE, c = FALSE, g = FALSE, t = FALSE), stringsAsFactors = FALSE)   # set
up a table of the iupac ambiguity codes and their possible translations into bases
ambinuc$a[c(2, 3, 6, 8:12)] <- TRUE       # set "a" TRUE for all valid replacements
ambinuc$c[c(1, 4, 6, 7, 9:12)] <- TRUE        # set "c" TRUE for all valid replacements
ambinuc$g[c(2, 4, 5, 7, 8, 10:12)] <- TRUE        # set "g" TRUE for all valid replacements
ambinuc$t[c(1, 3, 5, 7:9, 11, 12)] <- TRUE        # set "t" TRUE for all valid replacements
ambinuc$anzVar <- as.numeric(ambinuc$anzVar)       # convert number of possible replacements (anzVar) to type numeric

fasta <- choose.files(default = "", caption = "Select FASTA formatted sequences file for disambiguation") # open the
"open file" dialogue

seqn <- read.fasta(fasta, as.string = TRUE)       # read in the chosen file
seqn <- data.frame(cbind(seqnam = paste(">", names(seqn), sep = ""), seqn = seqn), stringsAsFactors = FALSE)    #
reformat the sequence data for further processing
seqn$seqn <- gsub(" ", "", seqn$seqn)       # remove whitespace from sequences

for(i in 1:nrow(seqn)) {        # repeat the calculations for all single sequences
  temp <- seqn$seqn[i]      # make a temporary working copy of the sequence
  if(sum(is.element(ambinuc$code, unlist(strsplit(temp, split = ""))))) == 0) {         # check if any IUPAC ambiguity
codes are found in the sequence
    if(exists("fertig") == FALSE) fertig <- seqn[i,] else fertig <- rbind(fertig, seqn[i,])       # if the sequence
doesn't contain IUPAC ambiguity codes, make a sequence copy in the final sequence table
```

```
  } else {     # if the sequence contains ambiguities, the following translations are conducted
    ersatz <- ambinuc[is.element(ambinuc$code, unlist(strsplit(temp, split = ""))), ]       # extract replacement
information available for the detected ambiguity codes
    ersetzen <- unlist(strsplit(gsub("[a, c, g, t]", "", temp), split = ""))      # extract all ambiguities
    ersetzen <- data.frame(cbind(ersetzen, anzVar = NA), stringsAsFactors = FALSE)       # prepare a table to hold
information about the detected ambiguities and the number of necessary translations
    ersetzen$anzVar <- as.numeric(ersetzen$anzVar)      # reformat the table column ersetzen$anzVar into type numeric
    for(j in 1:nrow(ersetzen)) ersetzen$anzVar[j] <- ambinuc$anzVar[ambinuc$code == ersetzen$ersetzen[j]]      # gather
information on the number of necessary replacements for each ambiguity detected in the current sequence
    kombi <- prod(ersetzen$anzVar)      # calculate the number of possible combinations of single base translations
    disambseq <- data.frame(matrix(nrow = kombi, ncol = 2))      # prepare a table to hold the disambiguated sequences
    colnames(disambseq) <- colnames(seqn)        # name the table columns with the original column names
    disambseq$seqn[1:kombi] <- temp         # fill in the original sequence in column disambseq$seqn
    disambseq$seqnam[1:kombi] <- paste(seqn$seqnam[i], rownames(disambseq), sep = "_")         # name the sequences with
the original sequence name with a suffix appended
    wdh <- kombi         # define wdh for control of the necessary number of repeats
    for(j in 1:nrow(ersetzen)) {        # loop to replace all single ambiguities found in the original current sequence
      wdh <- wdh/ersetzen$anzVar[j]       # define the number of repeats of each base in the current replacement group
      wdhwdh <- kombi/(wdh*ersetzen$anzVar[j])      # calculate the number of repeats for the replacement groups
      unambnuc <- rep(rep(names(which(t(ersatz[ersatz$code == ersetzen$ersetzen[j], ])[,1] == TRUE)), each = wdh),
wdhwdh)      # prepare a vector containing the necessary number of repeats of the replacement groups
      for(k in 1:length(unambnuc)) disambseq$seqn[k] <- sub(ersetzen$ersetzen[j], unambnuc[k], disambseq$seqn[k])
# loop to replace the current ambiguity with the predefined replacement
    }
    if(exists("fertig") == FALSE) fertig <- disambseq else fertig <- rbind(fertig, disambseq)       # append the
disambiguated sequences to the table holding the final sequence data
  }
}
newfasta <- paste(dirname(fasta), paste("/disambig_", basename(fasta), sep = ""), sep = "")        # create the filename
to store the disambiguated sequences
writeLines(paste(fertig$seqnam, fertig$seqn, sep = "\n"), newfasta)        # write the final sequences (disambiguated
and original unambiguous) into a new file named like the original file with the prefix "disambig" in the same folder as
the original file
```
